# Resveratrol Suppresses Epithelial-Mesenchymal Transition in GBM by Regulating Smad-Dependent Signaling

**DOI:** 10.1155/2019/1321973

**Published:** 2019-04-17

**Authors:** Yang Song, Yong Chen, Yunqian Li, Xiaoyan Lyu, Jiayue Cui, Ye Cheng, Tianyang Zheng, Liyan Zhao, Gang Zhao

**Affiliations:** ^1^Department of Neurosurgery, First Hospital, Jilin University, Changchun, Jilin 130021, China; ^2^Department of Medical Laboratory, Second Hospital, Jilin University, Changchun, Jilin 130041, China; ^3^Department of Histology and Embryology, College of Basic Medicine, Jilin University, Changchun, Jilin 130021, China

## Abstract

Glioblastoma (GBM) is the most common and malignant intracranial tumor in adults. Despite continuous improvements in diagnosis and therapeutic method, the prognosis is still far away from expectations. The invasive phenotype of GBM is the main reason for the poor prognosis. Epithelial-mesenchymal transition (EMT) is recognized as a participator in this invasive phenotype. Resveratrol, a natural plant-derived compound, is reported to be able to regulate EMT. In the present study, we used TGF-*β*1 to induce EMT and aimed to evaluate the effect of resveratrol on EMT and to explore the underline mechanism in GBM. Western blotting was used to detect the expression of EMT-related markers, stemness markers, and Smad-dependent signaling. Wound healing assay and transwell invasion assay were performed to evaluate the migratory and invasive ability of GBM cells. Gliosphere formation assay was used to investigate the effect of resveratrol on the ability of self-renewal. Xenograft experiment was conducted to examine the effect of resveratrol on EMT and Smad-dependent signaling* in vivo*. Our data validated that resveratrol suppressed EMT and EMT-associated migratory and invasive ability via Smad-dependent signaling in GBM cells. We also confirmed that resveratrol obviously inhibited EMT-induced self-renewal ability of glioma stem cells (GSCs) and inhibited EMT-induced cancer stem cell markers Bmi1 and Sox2, suggesting that resveratrol is able to suppress EMT-generated stem cell-like properties in GBM cells. Furthermore, we also showed the inhibitory effect of resveratrol on EMT in xenograft experiments* in vivo*. Overall, our study reveals that resveratrol suppresses EMT and EMT-generated stem cell-like properties in GBM by regulating Smad-dependent signaling and provides experimental evidence of resveratrol for GBM treatment.

## 1. Introduction

Glioblastoma is the most aggressive solid tumor and the most common primary brain tumor in humans [1]. Nowadays, the standard therapy for newly diagnosed glioblastoma is surgical resection in combination with radiotherapy and chemotherapy [2]. Even with continuous improvements in the treatment, the prognosis is still poor with a median overall survival of only 14.6 months after diagnosis [3].

An important property of GBM is that GBM cells tend to infiltrate and invade the surrounding normal brain tissue [4]. This invasive phenotype may lead to the difficulty of complete resection of GBM and recurrence after operation, which is the main cause of treatment failure [5]. Accordingly, it is very important for us to understand this invasive phenotype of GBM, which would be beneficial for the development of more sufficient therapeutic methods targeting GBM.

EMT is a complex biological process in which polarized epithelial cells undergo multiple biochemical changes and finally change into a mesenchymal phenotype [6, 7]. EMT plays a pivotal role in embryo formation, wound healing, and tissue remodeling. Nowadays it is also reported that EMT has much to do with cancer progression, invasion, and metastasis [8, 9]. The researches have indicated that EMT is considered as a mechanism causing this invasive phenotype of GBM cells [10]. Nevertheless, evidence of EMT in GBM has not yet been fully understood and still needs to be studied.

Growing evidence supports that EMT plays a key role in cancer stem-like properties, thus leading to drug resistance, relapse, and metastasis of many cancers [11–13]. The existing knowledge reveals that cancer stem cells (CSCs) are blamed for the invasion and resistance of the intracranial tumors [14]. EMT appears to impart not only metastasis ability but also cancer stem-like properties to tumor cells [15, 16]. EMT can be induced by many factors, including TGF-*β*1, EGF, FGF, Notch, and Wnt. TGF-*β*1 is a member of TGF-*β* superfamily which not only contributes to EMT during embryonic development but also induces EMT during the progression of tumors as well as metastasis [13, 17].

Resveratrol (Res) is a kind of polyphenol present in grapes, berries, peanuts, and wine [18–20]. It is proved that resveratrol has lots of bioactivities, including cardiovascular-protection and anti-inflammation and antiaging effect [21, 22]. Recent findings also indicate that resveratrol has strong antitumor potential against various types of cancer [23]. Besides, researchers have found the regulatory function of resveratrol on EMT [24], but it still needs investigating in GBM cells.

In this study, we first used TGF-*β*1 to induce EMT in GBM cells. Next, we clarified that resveratrol suppressed EMT, EMT-associated migration, and invasion and inhibited EMT-generated stem cell-like properties. We also confirmed that resveratrol suppressed EMT in xenograft experiments* in vivo*. Furthermore, our results showed that resveratrol exerted this inhibitory function* via* Smad-dependent signaling. Our study provided experimental evidence of resveratrol for glioma treatment and indicated Smad-dependent signaling as a potential therapeutic target of malignant glioma.

## 2. Results

### 2.1. TGF-*β*1 Induces EMT in GBM Cells

**Figure 1:**
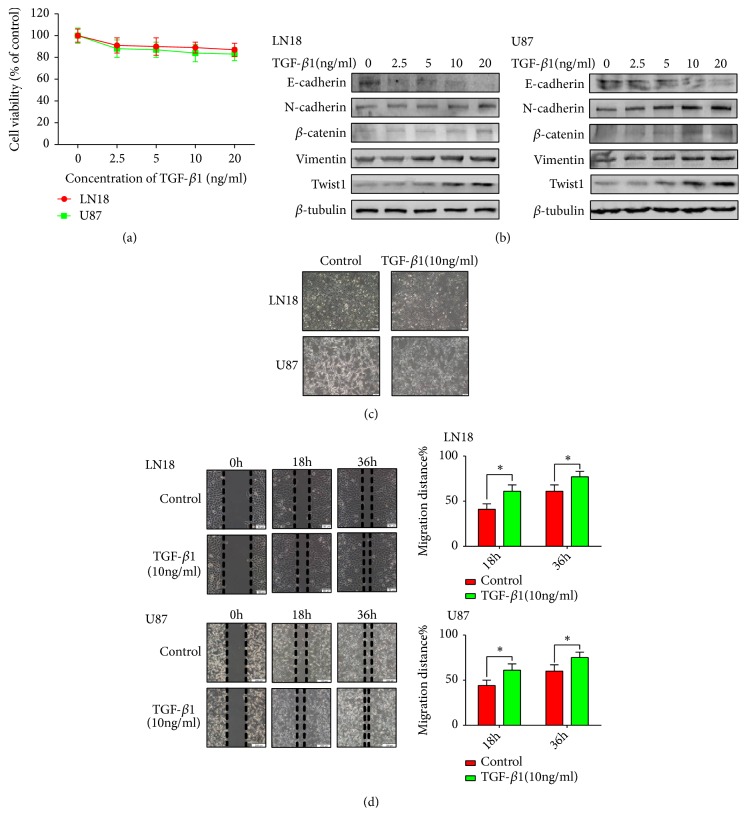
*TGF-β1 induces EMT in GBM cells.* (a) MTT assay of cell viability in LN18 and U87 cells following TGF-*β*1 treatment for 48 hours. (b) Western blotting showed that TGF-*β*1 induced downregulation of E-cadherin, upregulation of N-cadherin, *β*-catenin, Vimentin, and Twist1 expression in a dose-dependent manner. (c) Representative morphological images were photographed under Olympus microscope for LN18 (× 100 magnification) and U87 cells (× 100 magnification) treated with or without TGF-*β*1 (10 ng/ml) for 48 hours. (d) TGF-*β*1-induced EMT enhanced the migratory ability of LN18 and U87 GBM cells. Representative wound-healing images were photographed under Olympus microscope for LN18 (× 100 magnification) and U87 (× 40 magnification) GBM cells. The mean level of migration distance observed in three random fields for each condition was showed in histograms. Each independent experiment was repeated three times.

We first examined the effect of TGF-*β*1 on cell viability in LN18 and U87 GBM cells in order to exclude the influence of proliferation (Figure 1(a)). We discovered that TGF-*β*1 had almost no influence on cell viability. Thus, we chose a range concentration of TGF-*β*1 (0, 2.5, 5, 10, and 20 ng/ml) to continue the next experiment.

In order to illustrate the EMT phenomenon in GBM cells, our team examined the expression patterns of EMT-associated protein markers and important regulators. We discovered that the expression of epithelial markers E-cadherin decreased and the expression of mesenchymal markers N-cadherin and Vimentin increased after following TGF-*β*1 treatment in a dose-dependent manner in LN18 and U87 GBM cells (Figure 1(b)). *β*-catenin is an EMT-relative regulator, and Twist1 is recognized as critical transcription factor correlated with EMT. Consequently, our team went on a further investigation on the expression of *β*-catenin and Twist1. As a result, the protein level of *β*-catenin and Twist1 in GBM cells treated with TGF-*β*1 were also increased in a dose-dependent manner (Figure 1(b)). We clarified that 10 ng/ml TGF-*β*1 was efficient enough to induce this transformation of EMT-associated protein markers and important regulators, thus leading to EMT in GBM cells.

Later, our team observed the function of TGF-*β*1-induced EMT on the morphological changes of LN18 and U87 cells. We found that treatment with 10 ng/ml TGF-*β*1 for 48 hours transformed GBM cells into a more stretched and elongated morphology and altered their growth pattern to a more scattered one (Figure 1(c)).

To be continued, our team investigated the function of EMT on the migratory ability of LN18 and U87 cells. As expected, TGF-*β*1-induced EMT increased the migratory ability of LN18 and U87 cells in wound-healing assays (Figure 1(d)).

Taking these factors into consideration, our results reveal that TGF-*β*1 is able to induce EMT in GBM cells* in vitro*.

### 2.2. Resveratrol Suppresses EMT in GBM Cells

**Figure 2:**
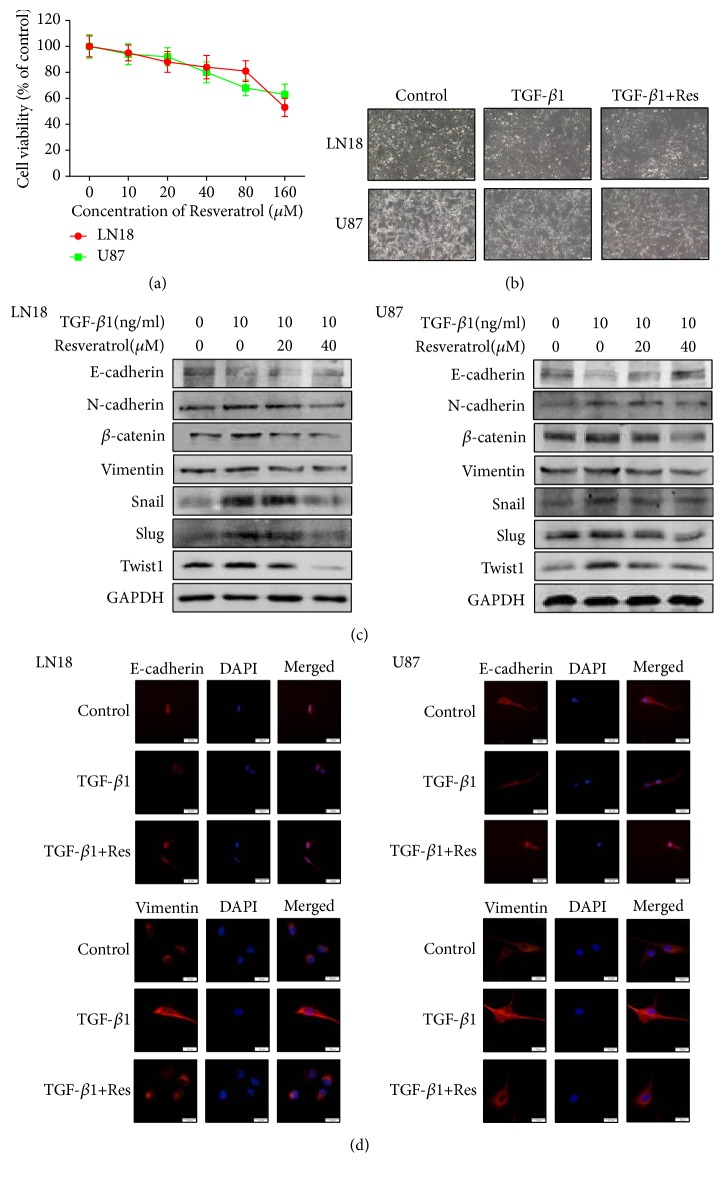
*Resveratrol (Res) suppresses EMT in GBM cells.* (a) MTT assay of cell viability in LN18 and U87 cells following resveratrol treatment for 48 hours. (b) Representative morphological images were photographed under Olympus microscope for EMT-induced LN18 and U87 cells treated with or without resveratrol for 48 hours (× 100 magnification). (c) Western blotting showed that resveratrol inhibited EMT-induced downregulation of E-cadherin and suppressed EMT-induced upregulation of N-cadherin, *β*-catenin, Vimentin, Snail, Slug, and Twist1 expression in a dose-dependent manner. All samples were analyzed in triplicate. (d) Immunofluorescence showed that resveratrol inhibited EMT-induced downregulation of E-cadherin and upregulation of Vimentin (× 400 magnification).

At first, our team assessed the effect of resveratrol on the proliferation of LN18 and U87 GBM cells. The data indicated that resveratrol had little effect on the cell viability below 40 *μ*M concentration (Figure 2(a)). Based on this situation, our team used 20 *μ*M and 40 *μ*M resveratrol for the following experiments.

To test the inhibitory effect of resveratrol on EMT, our team next examined the alteration of morphology after the resveratrol treatment in GBM cells.

As a consequent, there are no obvious morphological changes following treatment with resveratrol (Figure 2(b)), suggesting that resveratrol may not affect the morphology.

To clearly confirm the capacity of resveratrol to suppress EMT in GBM cells, our team continued to investigate the protein level changes of epithelial markers, mesenchymal markers, and important regulators. Our team discovered that the protein level of E-cadherin increased and the protein level of N-cadherin and Vimentin decreased in a dose-dependent manner following resveratrol treatment (Figure 2(c)). In consequence, resveratrol also inhibited the expression of *β*-catenin, Twist1, Snail, and Slug in a dose-dependency (Figure 2(c)). Furthermore, the results of immunofluorescence also showed that resveratrol suppressed EMT-induced downregulation of E-cadherin and upregulation of Vimentin (Figure 2(d)).

Our discoveries indicate that resveratrol can afford suppressing EMT in GBM cells via affecting the protein level of EMT-associated protein markers and important regulators.

### 2.3. Resveratrol Inhibits EMT-Associated Migration and Invasion of GBM Cells

Since resveratrol can inhibit EMT of GBM cells, our team continued to examine the function of resveratrol on EMT-associated migratory and invasive abilities in GBM cells. We employed wound-healing and transwell-based assays to properly evaluate the alteration of these properties in GBM cells.

**Figure 3:**
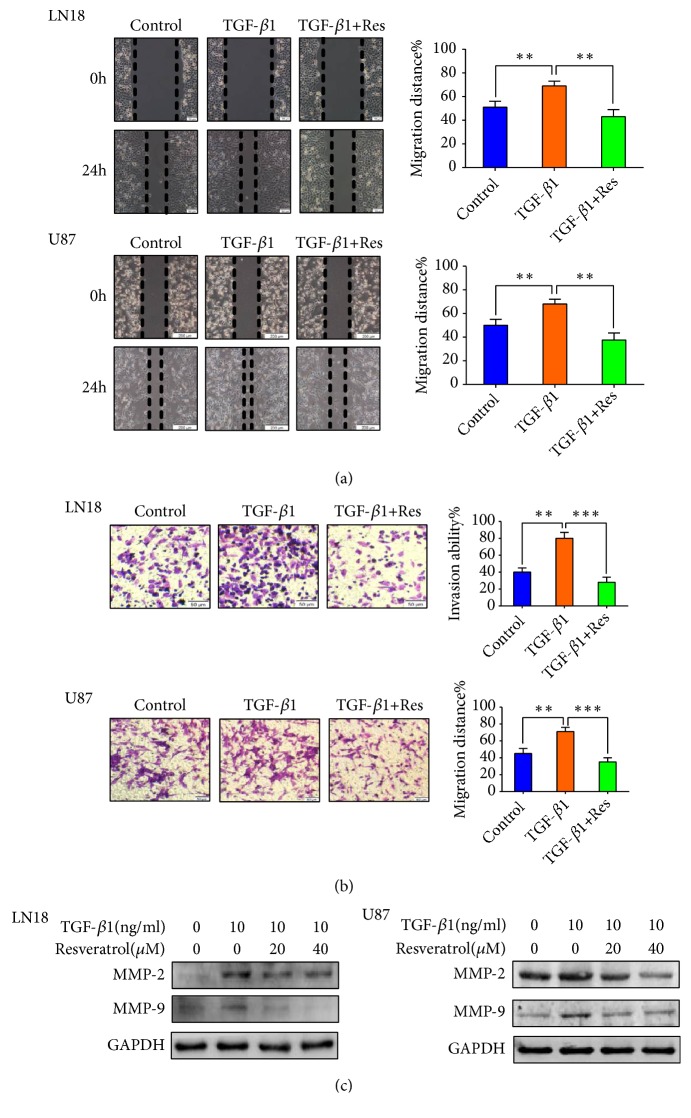
*Resveratrol (Res) inhibits EMT-induced migration and invasion of GBM cells.* (a) Resveratrol (40*μ*M) inhibited EMT-induced migratory ability in LN18 and U87 GBM cells. Representative wound-healing images were photographed under Olympus microscope for LN18 (× 100 magnification) and U87 (× 40 magnification) GBM cells. The mean levels of migration distance observed in three random fields for each condition were showed in histograms. (b) Resveratrol (40*μ*M) inhibited EMT-induced invasive ability in LN18 and U87 GBM cells. Representative transwell images were photographed under Olympus microscope for LN18 (× 100 magnification) and U87 (× 100 magnification) GBM cells. The mean levels of the numbers of cells counted in five random fields on each filter for each condition were showed in histograms. Each independent experiment was repeated three times. (c) Western blotting showed that resveratrol inhibited EMT-induced upregulation of MMP-2 and MMP-9 expression in a dose-dependent manner.

Our data exhibited that TGF-*β*1-induced EMT increased the migratory and invasive properties of LN18 and U87 cells. However, the enhanced tendency was suppressed after resveratrol treatment (Figures 3(a) and 3(b)). Furthermore, our team examined the EMT-associated protein matrix metalloproteinase-2 (MMP-2) and MMP-9. Our results showed that resveratrol inhibited EMT-induced upregulation of MMP-2 and MMP-9 expression (Figure 3(c)). Our findings indicate that resveratrol can remarkably inhibit EMT-associated migration and invasion of GBM cells.

### 2.4. Resveratrol Inhibits EMT-Generated Stem Cell-Like Properties in GBM Cells

**Figure 4:**
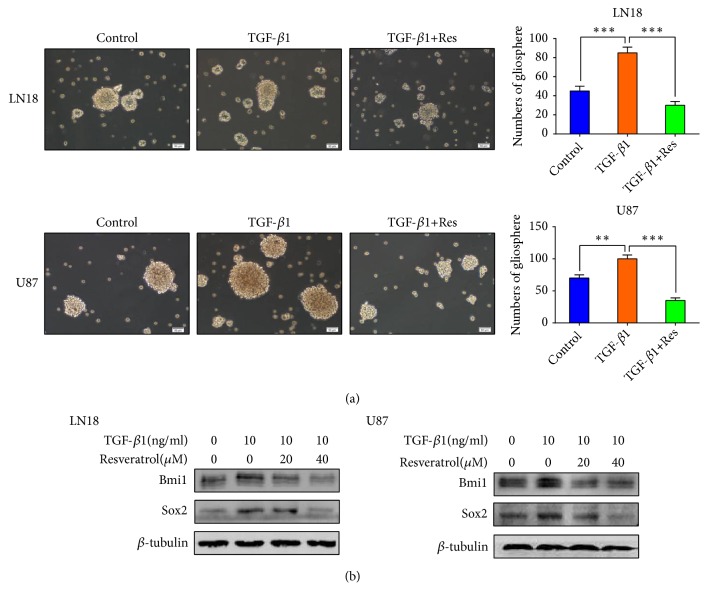
*Resveratrol (Res) inhibits EMT generated stem cell-like properties of GBM cells.* (a) Resveratrol (40*μ*M) inhibited EMT-induced gliosphere formation. Representative images of gliosphere were photographed under Olympus microscope (×100 magnification). The numbers of gliosphere in different treatment groups were showed in histograms. (b) Western blotting showed that resveratrol inhibited EMT-induced upregulation of stemness-related proteins expression in a dose-dependent manner. Each independent experiment was repeated three times.

EMT is tightly correlated with cancer stem cell-like properties. Since we had induced EMT in GBM cells, we further investigated the effect of EMT on acquisition of stem cell-like properties in GBM cells. A necessary feature of CSCs is the ability of self-renewal[25]. The ability of self-renewal of glioma stem cells (GSCs) was evaluated by using the secondary gliosphere formation assay* in vitro*. We tested the ability of secondary gliosphere formation of TGF-*β*1-induced LN18 and U87 cells and the results exhibited that the ability of secondary gliosphere formation was significantly raised (Figure 4(a)). We also examined the expression levels of CSCs protein markers, Bmi1 and Sox2. The data showed that EMT enhanced these CSCs protein markers expression in LN18 and U87 cells (Figure 4(b)).

We have validated that resveratrol is able to inhibit EMT in GBM cells, but whether resveratrol can inhibit EMT-generated stem cell-like properties in GBM cells remains unexplored. Therefore, we next assessed the stem cell-like properties in LN18 and U87 cells treated with resveratrol. We examined the effect of resveratrol on self-renewal ability by secondary gliosphere formation assay. The results showed that the ability of secondary gliosphere formation was apparently decreased following resveratrol treatment (Figure 4(a)). At the same time, resveratrol treatment also suppressed EMT-induced protein level of CSCs markers, Bmi1 and Sox2, in LN18 and U87 cells in a dose-dependency (Figure 4(b)).

Our discoveries validate that resveratrol can suppress EMT-generated stem cell-like properties in GBM cells.

### 2.5. Resveratrol Inhibits EMT by Regulating Smad-Dependent Signaling

There are several downstream effectors for the induction of EMT by TGF-*β*1, such as Smad-dependent signaling and Smad-independent signaling. The main signaling is through the phosphorylation of Smad proteins by TGF-*β* receptor serine/threonine kinase. Therefore, our team decided to estimate the function of resveratrol on this important Smad-dependent signaling.

**Figure 5:**
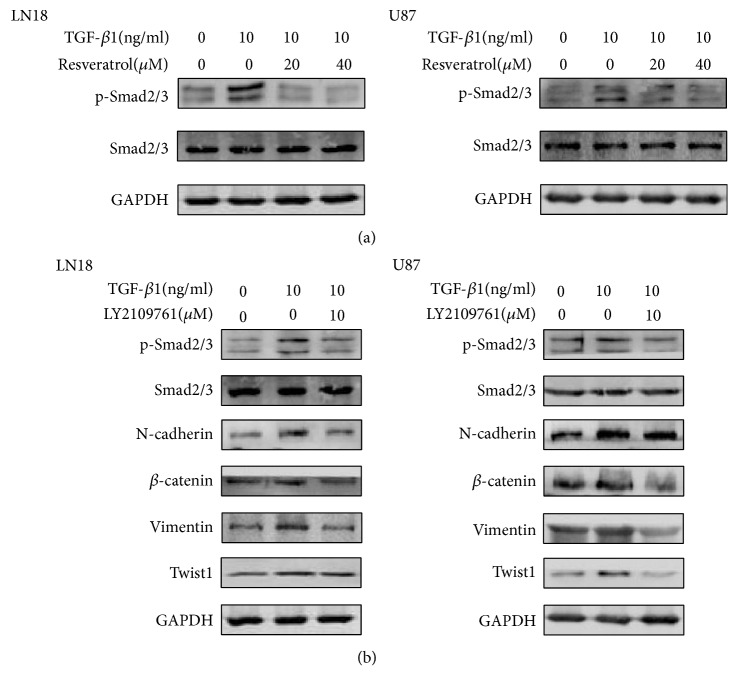
*Resveratrol inhibits EMT by regulating Smad-dependent signaling.* (a) Western blotting showed that resveratrol inhibited EMT-induced upregulation of p-Smad2(Ser465+Ser467)/p-Smad3(Ser423+Ser425) expression in a dose-dependent manner. (b) Western blotting showed that LY2109761 inhibited EMT-induced upregulation of p-Smad2(Ser465+Ser467)/p-Smad3(Ser423+Ser425) expression and inhibited EMT-induced upregulation of N-cadherin, *β*-catenin, Vimentin, and Twist1 expression. All samples were analyzed in triplicate.

Western blot analysis was conducted to investigate the phosphorylation status of protein Smad2 and Smad3. As a consequence, TGF-*β*1-induced EMT activated the phosphorylation status of protein Smad2 and Smad3 (Figure 5(a)). However, western blotting results clarified that phosphorylation status of Smad2 and Smad3 was seriously inhibited following resveratrol treatment (Figure 5(a)).

To further evaluate the inhibitory function of resveratrol on Smad-dependent signaling, our team selected LY2109761, a novel TGF*β*RI/II inhibitor to suppress the Smad-dependent signaling, and monitored the downstream effects (Figure 5(b)).

The data demonstrated that LY2109761 was effective in inhibiting Smad-dependent signaling. Moreover, EMT-induced upregulation of N-cadherin, Vimentin, *β*-catenin, and Twist1 was also inhibited by this inhibitor in LN18 and U87 GBM cells (Figure 5(b)).

These findings demonstrate that resveratrol inhibits EMT by regulating Smad-dependent signaling in GBM cells.

### 2.6. Resveratrol Inhibits EMT in U87 Xenografts in Nude Mice* In Vivo*

**Figure 6:**
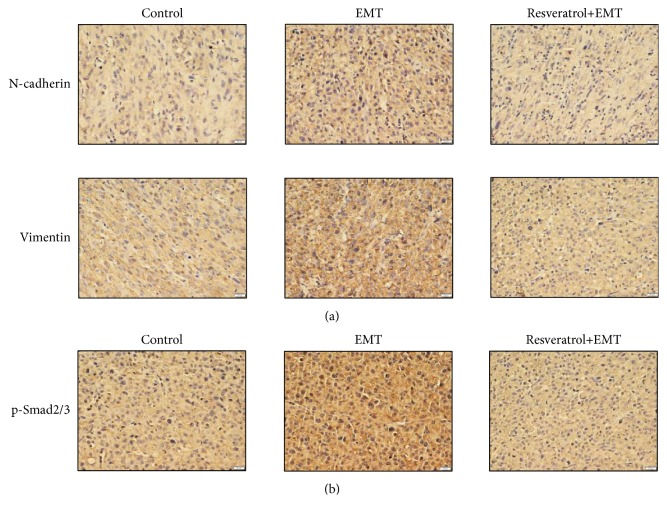
*Resveratrol (Res) inhibits EMT in U87 xenografts in nude mice in vivo.* Immunohistochemical detection showed that resveratrol (10 mg/kg) inhibited EMT-induced upregulation of N-cadherin, Vimentin, and p-Smad2/3 expression* in vivo*.

To further validate our results, our team used a U87 xenograft model so that we could clarify the inhibitory effect of resveratrol on EMT in nude mice in vivo. Immunohistochemical examination showed that TGF-*β*1-induced EMT increased the protein level of N-cadherin, Vimentin, and p-Smad2/3. Resveratrol administration significantly reduced the expression of N-cadherin, Vimentin, and p-Smad2/3 in xenograft tumors* in vivo* (Figures 6(a) and 6(b)).

**Figure 7:**
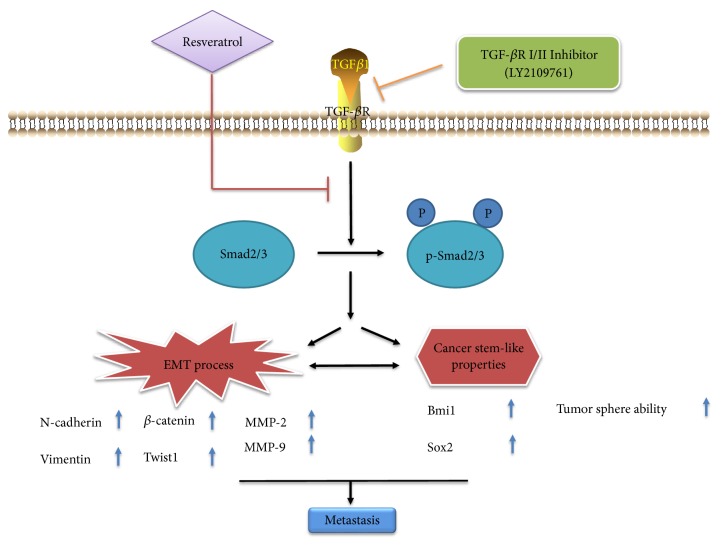
A working model depicts the inhibitory effect of resveratrol on EMT in GBM.

Taken together, our results gave an indication of the inhibitory effect of resveratrol on EMT.  Figure 7 sums up a potential work pattern depicting the inhibition of resveratrol on EMT phenomenon.

## 3. Discussion

The invasive phenotype of GBM cells is a main reason for the poor prognosis in patients. EMT is a pivotal procedure in cancer progression that is usually activated in the process of cancer invasion and metastasis [26, 27]. It is reported that EMT is closely connected with invasion and migration in many human tumors such as breast cancer [28] and lung cancer [29]. However, EMT phenomenon is a little bit complex and still need more exploration in GBM cells[30]. In epithelial cancers, epithelial tumor cells detach and spread through basement membrane (BM), which is a special type of extracellular matrix (ECM). Glioma cells spread through invading the ECM, which is a complex mixture of glycosaminoglycans, laminin, fibronectin, tenascin, nidogen, and fibrillar collagens [31, 32]. Many recent studies have shown that unlike what is previously believed, EMT is not a binary process and cells can adopt a hybrid epithelial/mesenchymal or a partial EMT phenotype [33, 34]. Cells transitioning between epithelial and mesenchymal phenotypes can adopt an intermediate state which has a mix of epithelial and mesenchymal traits [34]. EMT is always accompanied with increased expression of mesenchymal markers and decreased expression of epithelial markers. Some important regulators either act as transcription repressors by inhibiting epithelial-related genes or act as activator of mesenchymal genes, thus leading to the induction of mesenchymal phenotype and invasion in cancer cells. To our knowledge, GBM cells could undergo an EMT process which leads to a transformation from a less epithelial phenotype to a more mesenchymal one[10].

As we know, TGF-*β*1 has been widely used as a potent inducer of EMT in multiple experimental studies, including glioma cells [35, 36]. Besides, TGF-*β*1 also regulates various biological process in development and cancer, including cancer stemness [37]. In the present study, we applied TGF-*β*1 as a tool to induce EMT and seek for proper agent targeting EMT.

We validated that TGF-*β*1-induced EMT is able to decrease the protein level of epithelial marker E-cadherin and increase the protein level of the mesenchymal markers N-cadherin, Vimentin, and the important regulators Twist1 and *β*-catenin, in GBM cells. In addition, TGF-*β*1-induced EMT also caused the alteration of morphology and enhanced the migratory ability in GBM cells.

Since EMT seriously influences the prognosis of patients suffered from GBM, it is indispensable to find proper medicine targeting this phenomenon. It seems that resveratrol can bring us some hope and inspiration. Recent studies have proved that resveratrol inhibits EMT and suppresses invasion and metastasis in gastric cancer cells [24] and lung cancer cells [38]. Resveratrol was also proved to be able to inhibit GBM cell proliferation and increase cell mortality [39]. Researchers have also found that resveratrol can decrease the expression of MMP-2 [40].

However, whether resveratrol can also inhibit EMT in GBM cells has not been fully explored. In the present study, our team discovered that resveratrol is able to recover the protein level of epithelial marker and decrease the protein level of mesenchymal markers and crucial regulators, which thus suppressed EMT in GBM cells. Moreover, the inhibitory effect of resveratrol on EMT led to the inhibition of migration and invasion in GBM cells. Our results were further proved by immunoblot analysis, which showed that resveratrol suppressed EMT-induced upregulation of MMP-2 and MMP-9 protein level. As far as we know, it is clear that resveratrol inhibits EMT and EMT-associated migration and invasion in GBM cells for the first time.

EMT has been reported to play a critical role in tumor metastasis and recurrence, which have been shown to be tightly linked with the function of CSCs[41]. CSCs are a subpopulation of cells in a heterogeneous tumor featured by not only inducing tumor progression but also causing metastasis and recurrence of tumor [42]. CSCs have also been regarded as an important role leading to tumor migration and invasion [25]. The association of CSCs with EMT in cancer was recently established, as similarities in these two fields were noted for contributing to tumor recurrence, metastasis, and drug resistance.

More recently, evidence has emerged that the induction of EMT endows stem cell-like properties in differentiated mammary epithelial cells and breast cancer cells [15, 43]. It is also reported that the induction of EMT by TGF*β*-1 increases stemness properties in primary lung cancer cells [44]. Evidence seems to indicate that EMT can endow cancer cells not only with the migratory and invasive property, but also with stem cell-like properties.

A large body of evidence suggests that EMT and CSCs have similar functions. EMT and CSCs are closely connected with each other; both of them play crucial roles in the progression and metastasis of cancer. There is also an overlap regarding the stimuli that can induce the generation of EMT and CSCs[45]. EMT and CSCs are becoming highly relevant targets in anticancer drug discovery. Agents that inhibit the EMT process may serve as dual inhibitors by not only inhibiting the invasion of cancer cells but also suppressing EMT-generated stem cell-like properties. Researchers have found that resveratrol may act as a potential radiation sensitizer and enhance the effect of radiation on the stemness for GSC line SU-2[46].

As mentioned above, a growing body of evidence supports the close link between EMT and cancer stemness; however, an emerging notion has been raised. The recent studies have proposed the emerging concept of association between partial EMT and cancer stemness [47, 48] and a novel model that links a partial EMT with stemness [49]. Therefore, we investigated EMT-generated stem cell-like properties in GBM cells.

Importantly, our results showed that TGF-*β*1-induced EMT obviously enhanced the secondary glioshpere formation, suggesting that TGF-*β*1-induced EMT enhances self-renewal ability. Stemness of CSCs has been proved to be associated with massive cancer stem markers, such as Bmi1 [50] and Sox2 [51]. TGF-*β*1-induced EMT also increased the expression level of these cancer stem markers, suggesting that TGF-*β*1-induced EMT enhances the stemness of GSCs. These findings give us a revelation that a partial EMT can gain stem cell-like properties in GBM cells. Our results are consistent with the emerging concept that a partial EMT is more stem-like than a complete EMT [49, 52].

Continuously, our team investigated the effect of resveratrol on EMT-generated stem cell-like properties in GBM cells. Our results revealed that resveratrol apparently decreased EMT-induced secondary gliosphere formation and the expression level of cancer stem markers, suggesting resveratrol inhibits EMT-generated stem cell-like properties in GBM cells.

To be continued, we went on a further exploration on the potential mechanism of the inhibitory effect of resveratrol on EMT phenomenon. Recent studies showed that resveratrol is able to suppress EMT in colorectal cancer through TGF-*β*1/Smads signaling [53]. TGF-*β* combines with the receptors (TGF-*β*RI/II) to activate various downstream signaling, including Smad, MAPK and PI3K/Akt signaling. Among them, Smad-dependent signaling is a specific signaling which is closely related to the differentiation, invasion, and migration of cells and has been definitely proved to mediate TGF-*β*-induced EMT [54]. In the Smad-dependent signaling, TGF-*β* leads to the phosphorylation of Smad2 and Smad3 and facilitates the formation of Smad complex; then this Smad complex translocates into the nucleus and mediates EMT-related transcription [55, 56]. Our data showed that TGF-*β*1-induced EMT increased phosphorylation of Smad2 and Smad3 and thus activated the Smad-dependent signaling. Our results exhibited that resveratrol inhibited EMT-induced the phosphorylation of Smad2 and Smad3 in a dose-dependent manner, suggesting the function of resveratrol on EMT is related to Smad-dependent signaling.

In order to clearly clarify the effect of resveratrol on Smad-dependent signaling, our team suppressed the phosphorylation status of Smad proteins with LY2109761, a novel TGF*β*RI/II inhibitor. Lately, investigators have clarified LY2109761 has antitumor function as well as the ability to suppress EMT [53, 57]. As expected, suppressing the phosphorylation status of Smad proteins showed similar inhibitory function on the expression of mesenchymal markers and suppressed the expression of the important regulators in GBM. Based on the findings, our team validated that Smad-dependent signaling was definitely related to the inhibitory effect of resveratrol on EMT in GBM cells.

Our results were further validated in U87 xenograft tumor in nude mice* in vivo*. TGF-*β*1-induced EMT upregulated the protein level of N-cadherin, Vimentin, and p-Smad2/3, whereas this tendency was inhibited by resveratrol treatment in U87 xenografts, suggesting that resveratrol could inhibit EMT process by regulating Smad-dependent signaling* in vivo*.

Our findings provided potential possibility for resveratrol as an antiglioma medicine. Although the problem of resveratrol bioavailability and metabolism needs to be solved, many studies still tried to investigate the potential effect of resveratrol on cancer cells [53, 58, 59]. The nontoxic nature and antitumor effect make resveratrol a compelling candidate to GBM adjuvant therapies. Novel delivery methods such as resveratrol analogues (derivatives), nanoparticle formulations [60, 61], targeted delivery of mixed anticancer drugs, and direct injection (i.e., convection-enhanced delivery [62]) could potentially be used to achieve and maintain therapeutic doses in the brain.

## 4. Conclusions

To summarize, our discoveries validate that resveratrol can sufficiently suppress EMT, EMT-associated migration, and invasion and inhibit EMT-generated stem cell-like properties in GBM cells through affecting Smad-dependent signaling* in vitro.* We also confirm that resveratrol can inhibit EMT process* in vivo*. These facts may reveal an important use of resveratrol for the therapy of GBM and indicate Smad-dependent signaling as a therapeutic target for treating GBM, which provides crucial evidence for further experiment of resveratrol.

## 5. Materials and Methods

### 5.1. Reagents

Recombinant human TGF-*β*1 was obtained from Peprotech (USA). After dissolving in 10 mM citric acid, TGF-*β*1 was stored at −80°C. Resveratrol and LY2109761 were obtained from ApexBio and all dissolved in DMSO to acquire a storage liquid of 100 mM and 10mM concentration at −20°C.

### 5.2. Cell Culture

Human LN18 and U87 GBM cells were obtained from ATCC and were grown in Dulbecco's modified eagle medium (DMEM), supplemented with 10% fetal bovine serum (Biowest, South America Origin) and 100 U/ ml Penicillin-Streptomycin. The cells were tested and authenticated by testing short tandem repeats (STR) using PureLink® Genomic DNA Mini Kit in 2017. The cells were maintained at 37°C in an incubator with a controlled humid environment composed of 95% air and 5% CO_2_.

### 5.3. Gliosphere Formation Assay

According to previous literature, the self-renewal ability of tumor cells was determined by tumor sphere formation assay [63]. The primary glioshperes were obtained after culturing LN18 and U87 cells in Neurobasal medium added with 1%N2, 2%B27, 20ng/ml b-FGF, 20ng/ml EGF, 1% glutaMAX, 2*μ*g/ml heparin, and 100 U/ml Penicillin-Streptomycin for 7 days. Then the gliospheres were dissociated and seeded for secondary sphere formation. Following treatment with TGF-*β*1 or resveratrol for 7 days, the cells were observed and photographed under Olympus microscope. Each independent experiment was repeated three times.

### 5.4. Cytotoxicity Assay

MTT assay was used as the assessment of cell viability. LN18 and U87 cells were incubated with TGF-*β*1 or resveratrol for 48 hours after incubation in 96-well plates overnight. 20*μ*l MTT (5mg/ml) was added to each well and incubated for 4h. After discarding the supernatant, we added DMSO to each well. The absorbance was monitored at 570 nm wave length by use of a microplate reader (Bio-Rad, Hercules, CA, USA). Each independent experiment was repeated three times.

### 5.5. Immunofluorescence

LN18 and U87 GBM cells were plated onto polyL-lysine (Sigma) coated glass cover slips in DMEM with 10% FBS for 12 hours. GBM cells were washed with cold PBS, fixed with 4% paraformaldehyde for 30 min, permeabilized with 0.1% Triton X-100 for 15 min, and blocked in 5% BSA (Sigma) for 1 hour at room temperature. Then LN18 and U87 cells were immunostained with E-cadherin (1:100, Affinity, Rabbit pAb, AF0131) or Vimentin (1:100, CST, Rabbit mAb, #5741), at 4°C overnight. Subsequent visualization was performed with fluorochrome-conjugated secondary antibody (ZSGB-BIO, China) for 0.5 hours at room temperature in darkness, and the nuclei were counterstained with DAPI. The fluorescent signals were detected and photographed with a fluorescence microscope (Olympus IX51, Japan).

### 5.6. Western Blotting

The cells treated with indicated medicine were collected and lysed in RIPA Buffer (Beyotime Biotechnology, catalog number: P0013B) to get protein extracts. The level of total proteins was calculated by BCA (Beyotime Biotechnology, catalog number: P0010) method. Samples containing 30 *μ*g of total protein were loaded per well and were separated on SDS-PAGE and transferred by wet or dry transfer to PVDF membranes (Merck Millipore, pore size:0.45*μ*m,catalog number:IPVH00010) following incubation with indicated primary antibodies. Antibodies against E-cadherin (Affinity, Rabbit pAb, AF0131), N-cadherin (CST, Rabbit mAb, #13116), Vimentin (CST, Rabbit mAb,#5741), Snail (CST, Rabbit mAb, #3879 ), Slug (CST, mAb, #9585), *β*-catenin (CST, Rabbit mAb, #8480), Twist1 (Wanleibio, Rabbit, pAb, WL00997), MMP-2 (CST, Rabbit mAb #40994), MMP-9 (CST, Rabbit mAb #13667), Bmi1(CST, Rabbit mAb, #6964), Sox2 (CST, Rabbit mAb, #3579), p-Smad2 (Ser465+Ser467)/p-Smad3(Ser423+Ser425)(Wanleibio, Rabbit, pAb, WL02305), and Smad2/3 (Wanleibio, Rabbit, pAb, WL01520) were diluted in primary antibody dilution buffer (Beyotime Biotechnology, catalog number: P0023A) in indicated dilution rate according to their instructions, respectively. Anti-*β*-tubulin (ORIGENE, TA503129Z) and anti-GAPDH antibodies (ORIGENE, TA802519Z) were purchased from ORIGENE. After incubating with secondary antibodies (Boster, Wuhan, China, catalog number: BA1054 and BA1050) and ECL (Thermo Scientific Pierce™ ECL Western Blotting Substrate, 32106), and the membranes were scanned by using the Gel Imaging System from Sage Creation (Beijing). All samples were analyzed in triplicate.

### 5.7. Migration and Invasion Assays

The migration and invasion assays were performed as previously reported [64]. The migration ability of GBM cells was monitored by wound-healing assay. LN18 and U87 GBM cells were seeded to a 6-well plate at a density of 2 ×10^5^ cells/well. A scratch was made with a 10 *μ*l pipette tip in a confluent cell monolayer. The images of scratches were photographed at indicated time points under an inverted microscope. Each independent experiment was repeated three times.

The invasion capacity of GBM cells was monitored by using transwell (Corning)-based assay. GBM cells were trypsinized and seeded into the top chamber at a density of 5×10^4^cells per well in 200*μ*l serum-free medium. Cells were wiped away from the insert tops with cotton swabs after incubation at 37°C for 48 hours. Cells that invaded through the 8 *μ*m polycarbonate basement membrane were stained by using crystal violet. Cells were counted at randomly selected five fields of each well under a microscope. Each independent experiment was repeated three times.

### 5.8. Xenograft Experiment* In Vivo*

All animal experiments were approved by the Animal Ethics Committee of Jilin University. Xenograft experiment was performed as previously reported [4] and was improved. Six-week-old male nude mice (BALB/c-nu) purchased from Beijing Vital River Laboratory Animal Technology Co., Ltd., were housed in a sterile environment with a light/dark cycle of 12/12h and were allowed free access to food and water. Mice were subcutaneously injected in the right flank with 5×10^6^ U87 cells pretreated with or without TGF-*β*1 (10 ng/ml) in 100 *μ*l resuspended DMEM media. Tumor sizes were measured with a caliper and were calculated as 1/2×length×width^2^ in mm^3^. After the subcutaneous tumors reached an average size of 150 mm^3^, the tumor-bearing mice were divided into three groups. Mice in resveratrol groups were intraperitoneally injected with resveratrol (10 mg/kg body weight) once a day. Mice in control groups and TGF-*β*1 pretreated groups were intraperitoneally injected with DMSO in PBS once a day. Two weeks later, the mice were sacrificed and the tumors were harvested and fixed in 4% paraformaldehyde waiting for immunohistochemical examination.

### 5.9. Immunohistochemical Examination

Immunohistochemistry was used to examine the expression of N-cadherin in xerografted tumors. The harvested tumors were fixed in 4% paraformaldehyde for 24h. Four-micrometre thick sections were prepared from the paraffin-embedded blocks and antigenic retrieval was performed in citric acid buffer (pH 6.0). After cooling at room temperature, sections were treated with 3% hydrogen peroxide to block endogenous peroxidase activity. After incubation with blocking buffer (goat serum, MaiXin, China, KIT-9706) at room temperature for 20 minutes, sections were incubated with primary antibody N-cadherin (CST, Rabbit mAb, #13116), Vimentin (CST, Rabbit mAb,#5741), and p-Smad2 (Ser465+Ser467)/p-Smad3 (Ser423+Ser425) (Wanleibio, Rabbit, pAb, WL02305) at 4°C overnight. Then sections were incubated with second antibody (MaiXin, China, KIT-9706). After incubation with horseradish peroxidase-labeled streptavidin, antibody binding was visualized using 3,3′-diaminobenzidine (DAB), and the sections were counterstained with hematoxylin. The negative controls were sections stained without the primary antibody. Sections were evaluated and photographed at 400× magnification under microscope.

### 5.10. Statistical Analysis

The data were presented as mean ±standard deviation (SD). Comparisons between two groups were conducted using two-tailed unpaired Student's t-test. Experiments with more than two groups were compared by one-way ANOVA with Tukey's multiple comparison. The statistical significance was considered valuable at* P*<0.05. Statistical analyses were carried out with GraphPad Prism 6.0.

## Data Availability

The data used to support the findings of the study are included within the article.
